# ^18^F-Fluorodeoxyglucose Positron Emission Tomography and Computed Tomography With Magnetic Resonance for Diagnosing Adult-Onset Still's Disease

**DOI:** 10.3389/fmed.2020.544412

**Published:** 2020-10-22

**Authors:** Sara Bindoli, Paola Galozzi, Fabio Magnani, Laura Rubin, Cristina Campi, Andrea Doria, Diego Cecchin, Paolo Sfriso

**Affiliations:** ^1^Rheumatology Unit, Department of Medicine, Padova University Hospital, Padova, Italy; ^2^Nuclear Medicine Unit, Department of Medicine, Padova University Hospital, Padova, Italy

**Keywords:** AOSD, imaging, PET, MR, diagnosis

## Abstract

**Objective:** The objective of the study was to assess the advantages of ^18^F-fluorodeoxyglucose (FDG) positron emission tomography and computed tomography with magnetic resonance (PET/CT-MR) in diagnosing and monitoring patients with adult-onset Still's disease (AOSD).

**Methods:** Participants in this retrospective case-control study underwent whole-body ^18^F-FDG-PET/CT-MR imaging. All PET scans were qualitatively and semiquantitatively analyzed using standardized uptake values (SUVs) normalized to liver uptake, i.e., we calculated the ratio (SUVr) between the minimum, maximum, and mean SUVs for different organs and tissues and the mean SUV for the liver. Disease activity scores were assessed using Pouchot's criteria.

**Results:** Eighteen patients diagnosed with AOSD and 24 controls (non-AOSD patients diagnosed with solid tumors, excluding lymphomas) were considered. A total of 38 PET/MR and nine PET/CT scans were analyzed. AOSD patients had higher SUVr than controls. All SUVr differed significantly between the patient and control group for bone marrow, and for the spleen, the only difference lacking statistical significance concerned the ratio of the minimum SUV for spleen to the mean SUV for liver. Though limited in number, AOSD responders to therapy showed lower uptakes during the period monitored. No correlations were found between Pouchot's scores and SUVr.

**Conclusion:** Our data revealed higher spleen and bone marrow ^18^F-FDG uptakes on PET/CT and PET/MR images in AOSD patients than in controls. Together with clinical examinations and laboratory data, PET/CT and PET/MR seemed more reliable than Pouchot's score in assessing disease activity.

## Introduction

With an incidence of 0.16–0.62 per 100,000 population worldwide, adult-onset Still's disease (AOSD) is a rare multisystem inflammatory disease that usually affects young adults (1). Although the exact pathogenesis behind AOSD remains unknown, a combination of genetic and environmental factors seems to be involved (2).

AOSD is clinically characterized by daily high-spiking fever, evanescent maculopapular skin rash, arthritis, musculoskeletal involvement, sore throat, and hepatosplenomegaly (3). Cardiopulmonary manifestations and liver dysfunction are rare. Central nervous system (CNS) and renal involvement have been described in a few case reports (4). Typical laboratory findings include leukocytosis with neutrophilia, hyperferritinemia, high transaminases, and elevated acute-phase reactants, such as erythrocyte sedimentation rate (ESR) and C-reactive protein (CRP). Fautrel et al. proposed glycosylated ferritin (GF) as a diagnostic biomarker of AOSD because low concentrations have been noted (<20%) in several patients (5).

AOSD can be classified as belonging to one of three main patterns, depending on its clinical course: a monocyclic or self-limiting pattern characterized by a single systemic episode followed by a remission within a few months; an intermittent or polycyclic systemic pattern involving recurrent disease flares, with a complete remission taking a few years and a chronic articular pattern, usually associated with polyarthritis (6). As there are no specific diagnostic tests for AOSD, once infections, malignancies, autoimmune diseases, and drug reactions have been excluded, the diagnosis can be guided by Yamaguchi's and/or Fautrel's clinical criteria, which are currently the most widely used in clinical practice (7, 8).

There are no established guidelines for managing AOSD, and its treatment is sometimes challenging (9). IL-1β was recently identified as a crucial inflammatory mediator, and AOSD has been shown to respond significantly to IL-1β blockade (10, 11). It is unclear as yet whether prompt treatment with IL-1 inhibitors would offer the greatest benefit (12), but early diagnosis could facilitate the recognition and management of some of the most severe complications (13). X-rays at disease onset or during its acute phase have not proved useful to the diagnostic process. No radiological criteria for diagnosing AOSD are currently available.

New imaging techniques like positron emission tomography and computed tomography (PET/CT) and positron emission tomography with magnetic resonance (PET/MR) are beginning to play an important part in the diagnosis of numerous disorders, and in the monitoring of various autoimmune and autoinflammatory diseases. ^18^F fluoro-2-deoxyglucose (^18^F-FDG) is one of the most widely used positron-emitting radiopharmaceuticals. It is the isotope of an element that occurs naturally in organic molecules. PET was first used to detect malignancies because of the increased aerobic glycolysis of cancer cells. PET exploits this characteristic by using an ^18^F-FDG glucose analog as a tracer. This tracer becomes concentrated not only in malignant tissues, however, but also at sites of infection and inflammation (14, 15). PET imaging can be used for semiquantitative analyses by calculating the standardized uptake value (SUV), which is a mathematically derived ratio of tissue radioactivity concentration at a point in time C(T) and the injected dose of radioactivity per kilogram of the patient's body weight. In nuclear medicine, imaging has been taking giant steps forward in recent years, and the combination of PET and CT or MR, and PET-MR in particular, can now provide anatomical images with a high spatial resolution and excellent soft-tissue contrast (16).

The present study aimed to investigate the advantages and usefulness of ^18^F-FDG PET/CT-MR in establishing a diagnosis in AOSD patients, and monitoring their follow-up and treatment response. A secondary aim was to establish whether AOSD patients have a specific PET/CT-MR uptake pattern.

## Materials and Methods

### Patients

All AOSD patients referred to the Rheumatology Unit at the University of Padova were considered eligible for this study. AOSD was diagnosed on the basis of Yamaguchi's criteria. All patients had been steroid free for at least a month. Patients' disease activity was assessed using Pouchot's score (17), which is calculated by awarding one point for each of the following symptoms: fever, evanescent rash, pharyngitis, myalgia, pleuritis, pericarditis, pneumonia, lymphadenopathy, hepatomegaly or elevated transaminases, leukocyte count >1,500/mm^3^, and sore throat. Patients previously diagnosed with solid neoplasms were enrolled as controls to serve as a reference population with no inflammation or bone marrow proliferation in the semiquantitative analysis. Patients with lymphomas (known to involve a high uptake of both spleen and bone marrow) were therefore excluded from the normal dataset, as were cases of vasculitis, infectious diseases, endocarditis, and other hematological malignancies. All patients and controls underwent a clinical examination that included a review of their medical history and a physical examination, blood tests [white blood count (WBC), erythrocyte sedimentation rate (ESR), and C-reactive protein (CRP)], and instrumental investigations. Any past or present drug intake was recorded for all the patients. The study was performed in accordance with the principles of the Declaration of Helsinki. All participants gave their fully informed written consent at enrollment.

### ^18^F-Fluorodeoxyglucose Positron Emission Tomography and Computed Tomography With Magnetic Resonance

Patients and controls were prepared for the procedure according to a standard protocol that includes: fasting for at least 4–6 h, free hydration with water on the morning of the examination, and urination immediately before acquisition. They were informed about the risks and benefits of the procedure and assessed to identify any contraindications to their exposure to the high magnetic field (3T) or ionizing radiation. Tracer dosage was calculated on the basis of body weight (3 MBq/kg). The tracer was injected after checking a patient's blood glucose levels (glycemic threshold 200 mg/dl). Images were acquired ~45–60 min after injecting the tracer. In accordance with standard protocol, acquisitions were obtained from the top of the head to the knees, adopting a fixed scan duration of 5 min per bed position. The number of beds depended on the patient's height. DIXON-based MR images, including T1-weighted TURBO SPIN ECHO sequences (coronals in retained breath), T1-weighted VIBE FAT SAT sequences (transaxial in retained breath), and free-breathing (transaxial) T2-weighted TURBO SPIN ECHO sequences, were acquired for attenuation correction and localization of lesions. Attenuation correction of the PET images was done by interpolating the data acquired with the DIXON sequence, enabling the images to be divided into four different tissue components (water, fat, air, and pulmonary tissue). All four measurements were used to generate the densitometric map needed for attenuation correction of raw PET data. Each PET procedure lasted 45–60 min on average. The PET/CT-MR images were analyzed using Singovia (Siemens Healthineers) software, drawing regions of interest (ROIs) and volumes of interest (VOI) on spleen, bone marrow (BM), lymph nodes, pharynx, sublingual gland, and brown adipose tissue (BAT). We also examined the corresponding minimum, maximum, and mean SUVs (SUVmin, SUVmax, SUVmean), and calculated the ratio (SUVr) between the SUV for each ROI/VOI and the mean SUV for the liver. The SUVs for liver and spleen were obtained from ROIs of the same diameter drawn at the center of the hepatic and splenic parenchyma. The SUVs for bone marrow were obtained from VOIs placed over the central part of the vertebral bodies L3–L4–L5. The SUVs for lymph nodes were obtained from VOIs placed over the lymph node with the most intense FDG uptake.

### Statistical Analysis

Continuous variables were summarized as means ± standard deviations. The ^18^F-FDG-PET/MR and PET/CT data were first pooled for the statistical analyses, then a separate statistical analysis was run for the ^18^F-FDG-PET/MR data. The Mann–Whitney *U*-test was used to analyze statistical differences in the continuous SUVr variables (for spleen and bone marrow) between patients and controls. A value of *p* ≤ 0.05 was considered significant. The spleen-to-liver and BM-to-liver SUVr were calculated. Spearman's rank order correlation coefficient was used to ascertain the strength and direction of the relationship between the continuous SUVr variables and Pouchot's score in AOSD patients. The variables considered were Pouchot's score and the spleen-to-liver and BM-to-liver SUVr. *GraphPad Instat* version 3.00 was used for all statistical analyses.

## Results

Forty-two patients were included in this study, comprising 18 AOSD patients (eight males and 10 females) and 24 controls (12 males and 12 females) who underwent ^18^F-FDG PET/CT or PET/MR between 2008 and 2018. The two groups were comparable for sex and age; the mean age was 42.33 ± 18.80 years for the AOSD patients, and 44.6 ± 21.4 for the controls. We analyzed 14 ^18^F-FDG PET/MRs and 9 ^18^F-FDG PET/CTs performed in the AOSD group, and 24 ^18^F-FDG PET/MRs performed in the control group. Four patients underwent repeat procedures during the follow-up, involving four ^18^F-FDG PET/MRs and one ^18^F-FDG PET/CT.

The AOSD group had a variety of clinical features at the time of image acquisition, including fever, sore throat, joint pain, fatigue, maculopapular rash, and lymphadenopathy. The most frequent clinical symptoms were fever (max 39°C, 13/18 patients), rash (8/18), sore throat (4/18), myalgia (3/18), and joint pain (2/18). Table 1 lists the number of exams performed for each patient, the patients characteristics, the Pouchot's score at the time of PET acquisition, and the therapy they were undertaking.

**Table 1:** Characteristics of adult-onset Still's disease (AOSD) patients, main areas of uptake represented at positron emission tomography (PET) scans, and type of therapy undertaken at time of scan.

**Patient number**	**PET-computed tomography magnetic resonance (CT/MR) scans performed for each patient (total n = 23)**	**Pouchot's score**	**Main areas of organ uptake**	**Therapy**	**Supposed AOSD pattern**
1	1	3	LN, BM, spleen	Antibiotics	Polycyclic
	2	3	LN, (spleen equal as liver)	Anakinra	
	3	1	No uptake	Anakinra	
2	1	2	BM	na	Monocyclic
3	1	na	BAT	Corticosteroids	Monocyclic
4	1	4	BAT, LN	Anakinra	Polycyclic
5	1	1	Spleen, LN, BAT	Acetamynophen	Monocyclic
6	1	3	LN, BM	Acetamynophen	Polycyclic
	2	1	No uptake	Corticosteroids	
7	1	na	No uptake	na	Monocyclic
8	1	4	BM, spleen	Indomethacin	
9	1	3	BM	Corticosteroids/anakinra	Polycyclic
10	1	5	LN	Corticosteroids	Polycyclic/MAS
	2	6	LN, BM, spleen	Anakinra/corticosteroids/ cyclosporin	
11	1	na	No significant uptake	na	Monocyclic
12	1	3	BM	na	Monocyclic
13	1	2	BM	na	Polycyclic
	2	na	LN	Anakinra	
14	1	3	No significant uptake	Anakinra	Polycyclic
15	1	6	BM, LN, liver	Corticosteroids/cyclosporin	Polycyclic/MAS
16	1	na	Musculoskeletal diffuse uptake	Anakinra	Chronic articular
17	1	5	Spleen	na	Polycyclic
18	1	3	BM, liver	na	Monocyclic

*Legend: na, not available/no therapy; BAT, brown adipose tissue; BM, bone marrow; LN, lymph nodes*.

### Characterization of the Organs/Tissues Affected in AOSD Patients and Controls

Tracer uptake in other organs/tissues was considered pathological when higher than the liver uptake. A cutoff was set by adding the standard deviation multiplied by 2 to the mean ratio between the control group's SUVmax for the BM and SUVmean for the liver. Ratios of SUVmax for an organ/tissue to SUVmean for the liver that exceeded 2.09 identified significant areas of uptake in the organs/tissues considered.

Table 2 lists the organs/tissues affected in AOSD patients and controls based on the ^18^F-FDG PET/CT-MR and ^18^F-FDG PET/MR scans.

**Table 2:** Organ involvement in the AOSD patients and in the controls according to ^18^F-fluorodeoxyglucose-PET/MR-CT and ^18^F-PET/MR alone and the SUV values for each area.

	**SUVmin organ/liver**	**SUVmax organ/liver**	**SUVmean organ/liver**	**N of PET/CT or PET/MR with SUV max ratio **> =** 2.09**
**Affected organs according to PET/CT-MR in 18 AOSD patients (total 23 PET)**				
Spleen	0.8 ± 0.32	1.40 ± 0.41	1.07 ± 0.34	2/23
BM	0.91 ± 0.29	2.3 ± 0.75	1.36 ± 0.48	11/23
LN	2.00 ± 1.25	4.79 ± 2.87	3.15 ± 1.95	9/23 ^*^
Pharynx	2.89 ± 1.38	6.94 ± 3.61	3.80 ± 1.33	8/23
BAT	3.34 ± 2.98	8.34 ± 7.42	5.01 ± 1.12	3/23
SL glands	1.52 ± 0.88	3.65 ± 2.1	2.48 ± 1.5	9/23
**Affected organs according to PET/MR (only) in AOSD patients**				
Spleen	0.84 ± 0.36	1.41 ± 0.45	1.1 ± 0.40	1/14
BM	0.93 ± 0.30	2.39 ± 0.81	1.40 ± 0.51	7/14
**Affected organs according to PET/MR in 24 controls**				
Spleen	0.61 ± 0.14	1.05 ± 0.23	0.81 ± 0.16	0/24
BM	0.53 ± 0.14	1.34 ± 0.36	0.75 ± 0.20	1/24
LN	*Na*	*Na*	*Na*	
Pharynx	1.74 ± 0.46	4.31 ± 1.11	2.73 ± 0.78	4/24
BAT	*na*	*Na*	*Na*	
SL glands	1.21 ± 0.42	2.98 ± 1.05	1.86 ± 0.62	12/24

*The number of scans with an SUVmax organ-to-liver ratio ≥2.09 (representing high-uptake areas) are listed*.

*BAT, brown adipose tissue; BM, bone marrow; LN, lymph nodes; SL, sublingual; SUV, standardized uptake value*.

* *Lymph nodes were distributed in the abdominal region (five patients), head and neck district (two patients), axillary lymph nodes (one patient), and mediastinum (one patient)*.

**Figure 1:**
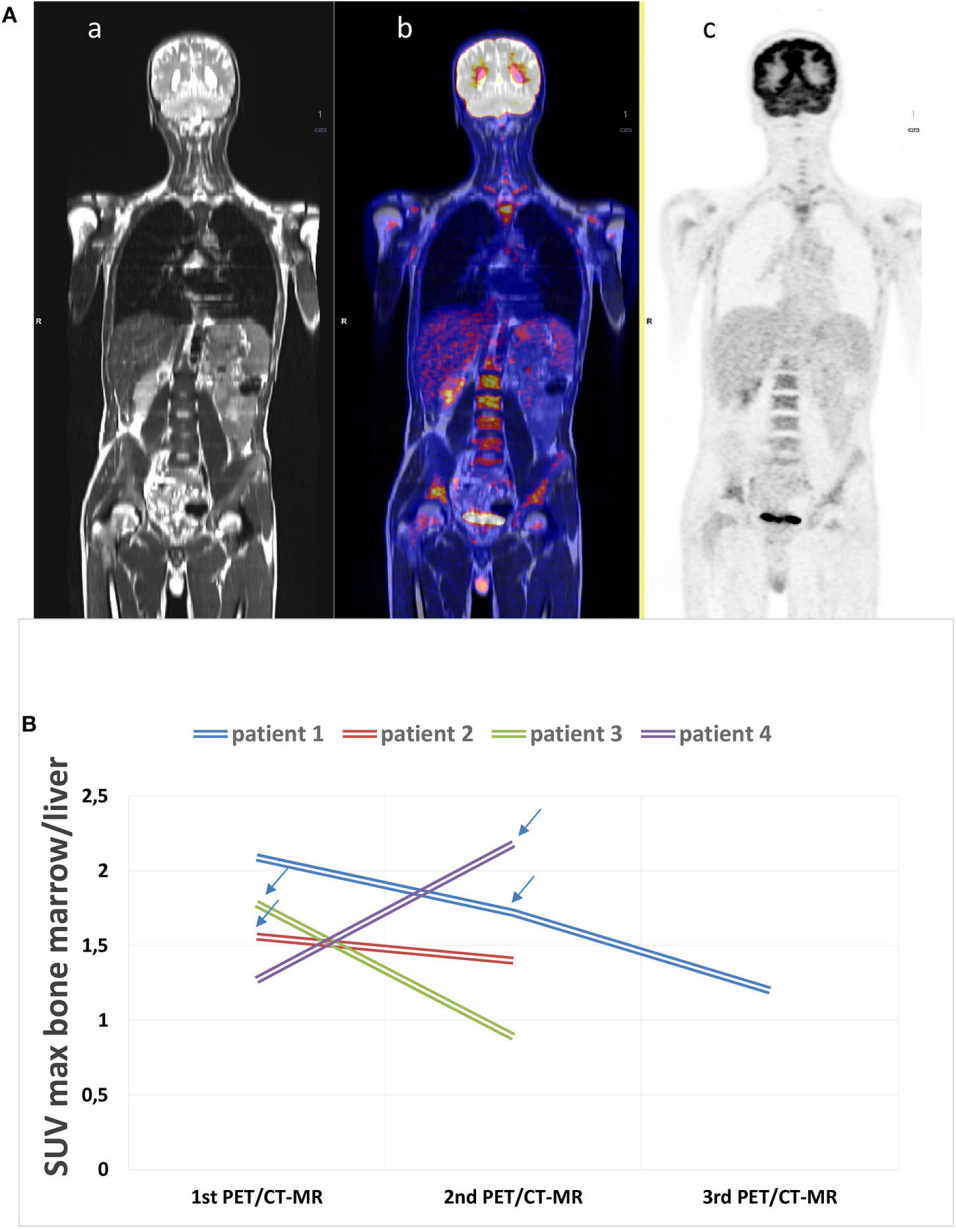
**(A)** Example of an ^18^F-fluorodeoxyglucose (^18^F-FDG) positron emission tomography with magnetic resonance (PET/CT-MR) scan at disease onset. (a) MR whole-body image, T1 turbo spin echo, coronal (WB_t1_tse_cor), (b) fused image, and c) PET acquisition image with attenuation correction (PET_AC). This scan shows a diffusely increased FDG uptake in the bone marrow (SUVmax 4.12), in the spleen (SUV max 3.13), and in the submandibular lymph nodes (SUVmax 5.61). The patient was diagnosed with adult-onset Still's disease (AOSD) and started therapy with glucocorticoids after acquiring the images. **(B)** ^18^F-FDG PET/CT-MR was repeated in four patients. The arrows state the moment IL-1 inhibitory therapy was started during the follow-up showing the decrease in bone marrow uptake.

All 23 PET/CT-MR (100%) scans of AOSD patients revealed sites of focal ^18^F-FDG uptake. A high, homogeneous accumulation was apparent in the spleen, BM, lymph nodes, pharynx, salivary glands, and BAT. The spleen, lymph nodes, and BM exhibited ^18^F-FDG uptake on the PET/MR scans obtained in AOSD patients (Figure 1A).

There were significant differences in the SUVr for the spleen and BM between the two groups based on the ^18^F-FDG PET/CT-MR and PET/MR images (Table 3). The *p*-value for each SUVr for BM was <0.001. For the spleen, the only difference that was not significant concerned the ratio between the SUVmin for the spleen and the SUVmean for the liver uptake.

**Table 3:** Comparison of the uptakes by the spleen/liver and bone marrow/liver according to ^18^F-FDG PET/CT-MR and to ^18^F-FDG-PET/MR alone in the two groups.

	**AOSD**			**Controls**			**Mann-whitney** ***U*****-test**		
	**SUVmin**	**SUVmax**	**SUVmean**	**SUVmin**	**SUVmax**	**SUVmean**	***p*-value SUVmin**	***p*-value SUVmax**	***p*-value SUVmean**
**18F-FDG PET/CT-MR**									
Spleen/liver	0.8 ± 0.32	1.40 ± 0.41	1.07 ± 0.34	0.61 ± 0.14	1.05 ± 0.23	0.81 ± 0.16	0.18	**0.0005**	**0.0013**
BM/liver	0.91 ± 0.29	2.3 ± 0.75	1.36 ± 0.48.	0.53 ± 0.14	1.34 ± 0.36	0.75 ± 0.20	**<0.0001**	**<0.0001**	**<0.0001**
**18F-FDG PET/MR**									
Spleen/liver	0.84 ± 0.36	1.41 ± 0.45	1.1 ± 0.40	0.61 ± 0.14	1.05 ± 0.23	0.81 ± 0.16	0.076	**0.0125**	**0.0093**
BM/liver	0.93 ± 0.30	2.39 ± 0.81	1.40 ± 0.51	0.53 ± 0.14	1.34 ± 0.36	0.75 ± 0.20	**<0.0001**	**<0.0001**	**<0.0001**

*BM, bone marrow; SUV, standardized uptake value. In bold, significant p value (<0.05)*.

### Correlations Between Spleen-to-Liver or BM-to-Liver SUVr and Pouchot's Score

The mean disease activity calculated with Pouchot's score in the AOSD patients was 2.61 ± 1.85. No significant correlations emerged between the spleen- or BM-to-liver SUVr and Pouchot's score in any of the AOSD patients (see Supplementary Table 1).

### Repeat ^18^F-Fluorodeoxyglucose Positron Emission Tomography and Computed Tomography With Magnetic Resonance Procedures During the Follow-Up Period

Four of the AOSD patients underwent PET/CT-MR more than once during the follow-up. Three patients had two scans, and one patient had three. This last patient underwent PET-MR twice and PET-CT once. Figure 1B shows the results of PET/CT-MR in the four patients, with variations in the SUVs (minimum, maximum, and mean) for the BM. Two of the patients showed an improvement in their later ^18^FDG PET/CT-MR images, with a generally lower uptake in the spleen and BM, which was consistent with their markedly improved clinical picture. These patients were taking anakinra during the follow-up. The other two patients showed an increase in uptake and worsening clinical picture due to a lack of response to steroids and non-steroidal anti-inflammatory drugs (NSAIDs). After the scans, the patients in therapy with anakinra showed a marked clinical improvement.

## Discussion

AOSD is typically diagnosed by exclusion, based on clinical findings, and numerous biochemical, microbiological, histological, and imaging tests. Yamaguchi's and Fautrel's criteria (7, 8) are currently the most widely used in clinical practice to establish AOSD diagnosis once neoplastic disorders and infections have been ruled out. Though no specific imaging tools for the clinical assessment of AOSD have been identified as yet, our study underscores how PET/CT-MR can facilitate the differential diagnosis from other diseases resembling autoimmune or autoinflammatory disorders, such as systemic lupus erythematosus (SLE), sarcoidosis, or IgG4-related disease (18). Neoplastic diseases usually present with specific symptoms related to the organ/tissue involved, and are generally accompanied by weight loss and rising levels of neoplastic markers, and first-level imaging (X-ray, ultrasound echotomography, gastrointestinal endoscopy, CT and MR) is often consistent with the suspected disease. Other investigations may be needed to exclude hematological disorders (leukemias, lymphomas). AOSD can be differentiated from hematological malignancies by the absence of B symptoms (e.g., night sweats, weight loss), while the presence of odynophagia, sore throat, and typical serum biomarkers (e.g., increased ferritin and low glycosylated ferritin) are pathognomonic expressions of AOSD (12). Although hematological malignancies may have similar tracer uptake patterns to those seen in AOSD, the ^18^F-FDG uptake in BM in patients with lymphomas is often multifocal, while it is usually homogeneous in AOSD (19). A complete blood workup and examinations such as lymph node biopsy, flow cytometry, and immunohistochemistry are often used to facilitate the diagnostic process because fluoro-2-deoxyglucose uptake is not specific to cancer cells. In fact, aerobic glycolysis increases in activated macrophages and fibroblasts, too, reflecting an inflammatory state. In this context, ^18^F-FDG PET/CT may help distinguish an infection or hyperinflammatory condition from an underlying neoplasm, and as it can reveal the extent of a lesion, it can also orient toward the most appropriate clinical approach (19). When specific serum markers or imaging are blurred and symptoms are misleading, distinguishing between AOSD and other conditions can still be quite a challenge. The main issue concerns patients with fever of unknown origin (FUO). A recent meta-analysis found PET/CT the best nuclear imaging test in patients with FUO, as it revealed the origin of the symptom in approximately 60% of cases with inconclusive basic laboratory test and anatomical imaging results (20). In a large prospective study on 240 patients with FUO and inflammation of unknown origin (IUO), ^18^F-FDG-PET/CT contributed remarkably to ameliorating the patients' clinical management (21).

When it comes to rheumatic diseases, ^18^F-FDG PET/CT generally provides sensitive, but non-specific information on active inflammatory lesions. The distribution pattern of inflammatory foci sometimes suggests an accurate early diagnosis and follow-up, as in cases of rheumatoid arthritis (RA), spondyloarthritis (SpA), polymyalgia rheumatica (PMR), relapsing polychondritis, large-vessel vasculitis (LVV), poly-dermatomyositis, or in autoinflammatory conditions like Schnitzler syndrome—and AOSD (22, 23). Patients with chronic inflammatory and autoimmune diseases (especially sarcoidosis, SLE, SpA and RA) often have PET/CT images comparable with those obtained in cases of AOSD (24), but particular sites of uptake can be expected in AOSD subjects. When Yamashita et al. compared FDG accumulation in AOSD as opposed to RA and SpA patients, all the AOSD patients, but <70% of the RA and SpA patients had a significant FDG accumulation in BM. SUVmax for the spleen was higher in AOSD (90.1%, score 3.20 ± 1.32) than in RA (50%, score 1.44 ± 0.63) or SpA (66.7%, score 1.67 ± 0.48), reflecting the systemic involvement in the case of AOSD (22).

The present study on a large series of ^18^F-FDG PET/CT-MR in 18 AOSD patients confirmed the value of such imaging methods in preventing misdiagnoses and revealing disease activity throughout the body. In our patients, ^18^F-FDG uptake accumulated mainly in the BM, spleen, and multiple lymph nodes, consistently with other reports in the literature. A diffuse uptake in these areas (18) is typical of AOSD because the natural pathophysiology of the disease involves macrophages and neutrophils as pivotal mediators of innate immunity. The first report on the use of PET/CT in a patient with suspected AOSD showed an intense uptake in liver and spleen that was suggestive of a malignancy because the spleen uptake was borderline between the SUV threshold used to distinguish between benign and malignant conditions. The patient's clinical features and laboratory test findings pointed to a diagnosis of AOSD, however, ^18^F-FDG PET/CT can be helpful in the diagnostic work-up provided that it is supported by clinical signs and symptoms (25, 26). Although the abovementioned areas of uptake (BM, spleen, and lymph nodes) are typical of systemic AOSD, other less commonly affected regions may come to light. In a study on 27 patients with AOSD, Jiang et al. also found radiotracer accumulation in the skin and joints (19). Similarly, Roy et al. detected elevated FDG levels in the joints of a 28-year-old AOSD patient (27). It is therefore conceivable that ^18^F-FDG-PET/CT-MR could help distinguish between systemic AOSD and the chronic articular pattern of this disease.

De Graaff et al. reported a marked uptake of ^18^F-FDG in the carotids and large vessels of the legs in a patient with AOSD (28). Ahn et al. reported a higher uptake in the sacroiliac joints and the wall of the rectosigmoid colon in a patient with AOSD and concomitant pseudomembranous colitis (29). Dong et al. retrospectively examined 744 patients with FUO and discovered that 26 were cases of AOSD (according to Yamaguchi's criteria). Tracer uptake was mainly in BM (100%) and spleen (96.15%). Lymph nodes of the neck, axilla, mediastinum, and hilum were also involved (up to 10 nodes, bilaterally). The general distribution of the radiotracer helped to rule out malignancies, and AOSD was confirmed on targeted biopsies (BM and lymph nodes) (24). An increased tracer uptake in BAT, pharynx, and sublingual glands has occasionally been reported. Taken together, the data emerging from the abovementioned literature suggest that AOSD is like a “mosaic” of underlying autoinflammatory foci reflecting the hypercytokinemia of this systemic disorder.

In our study sample, 34.7% of the patients had tracer uptake in the pharynx and 39.1% in the sublingual glands. Findings in the controls were quite similar, with 16.6% showing an increased uptake in the pharynx and 50% in the sublingual glands, so such a picture is not exclusive to AOSD. Analyzing the uptake in our patients' and controls' pharynx and salivary glands revealed no significant differences. The uptake in the controls might have been due to a non-specific inflammatory process or oral/upper respiratory tract infections. Tracer uptake in BAT was detected in 13.04% of our AOSD patients, but not in any of the controls. This type of uptake is generally considered non-specific.

Regarding laboratory tests, only a few studies investigated the correlation between SUVmax and laboratory biomarkers. Yamashita et al. found a relationship between lactate dehydrogenase and spleen SUVmax, but no other associations (22). Another retrospective study reported that visual grade of both spleen and BM correlated positively with ESR and leukocyte counts in at least 90% of the patients considered (30). Analyzing our data revealed no correlations between SUVmax and patients' disease activity based on their Pouchot's scores. This may have been due to the unreliability of the scoring system or to the small size of our sample (one of this study's limitations).

Our study is the first, to our knowledge, to have examined the value of ^18^F-FDG PET/MR in AOSD diagnostics. The procedure seems to overcome the limits of PET/CT and should theoretically be preferable because it enables a more specific assessment of the head and neck, prostate, brain, cardiovascular system, and musculoskeletal images (31). The advantages of PET imaging for the purposes of monitoring the effects of treatment are particularly evident in patients receiving biological agents or conventional synthetic disease-modifying antirheumatic drugs, as clinical and laboratory data are often insufficient for detecting any improvement in disease symptoms (32, 33). In our cohort, only four AOSD patients had repeat imaging procedures. One had PET/CT twice, and four had PET/MR twice (in three cases) or three times (in one). Two of the patients showed a lower uptake on repeat imaging that was consistent with their clinical picture after taking the anti-IL-1 receptor anakinra. Other two patients showed an increased uptake and worsening clinical picture after treatment with steroids and NSAIDs. They were then switched to anakinra 100 mg/days subcutaneously, leading to a significant clinical improvement. There are currently only a few published reports on the use of ^18^F-FDG-PET/CT for monitoring response to therapy (34, 35), so further studies on larger cohorts of AOSD subjects will be needed to establish whether PET/MR could be useful in assessing disease activity in patients on anti-IL-1 therapy. Although only four patients in our sample underwent repeat procedures, our preliminary findings indicate that PET/MR imaging enables a correct assessment of disease activity (consistent with clinical findings) and appropriate management of a patient's treatment. If PET/MR is repeated every 12 months for at least 2 years, the degree of uptake may reveal whether the disease is in remission or not when laboratory tests or clinical findings are unclear.

## Conclusion

Analyzing our study data confirmed that AOSD patients had a higher ^18^F-FDG uptake on PET/CT-MR images than the controls. The spleen, BM, and lymph nodes seem to be the pivotal areas of tracer uptake in patients with AOSD and may reflect disease activity. Since responders to anti-IL-1 had a lower uptake on repeat imaging, ^18^F-FDG PET/CT-MR in association with clinical findings seems to be more reliable than the Pouchot's score for the purpose of assessing disease activity. That said, large-scale studies will be needed to establish whether PET/MR can be used to identify patients who will respond to therapy and routinely adopted to assess disease remission—especially when clinical and laboratory findings are uncertain. Overall, our results confirm that PET/MR and PET/CT are effective in assessing disease activity in AOSD and in guiding treatment decisions.

This study suffers from the limitations of a small sample size (as concerns the patients who repeated the imaging procedure) and its retrospective nature.

Finally, it is worth bearing in mind that ^18^F-FDG PET/MR offers an advantage over PET/CT in terms of its safety and repeatability, especially in young people and fertile women.

## Data Availability Statement

The raw data supporting the conclusions of this article will be made available by the authors, without undue reservation.

## Ethics Statement

The studies involving human participants were reviewed and approved by the Ethics Committee of the Padua University Hospital. The patients/participants provided their written informed consent to participate in this study.

## Author Contributions

SB analyzed the data, prepared the tables, and designed and wrote the manuscript. FM performed and analyzed the PET/CT-MR scans and drafted the manuscript. LR contributed to recruiting patients and drafted the manuscript. CC supported the statistical and data analysis. AD revised the manuscript. PG critically examined and revised the manuscript. DC analyzed the PET/CT-MR scans, and critically examined and revised the manuscript. PS revised the manuscript, participated in the data analysis, and was responsible for financial support. All authors approved the final manuscript.

## Conflict of Interest

The authors declare that the research was conducted in the absence of any commercial or financial relationships that could be construed as a potential conflict of interest.
