# Supplementary material for: 18F-Fluorodeoxyglucose Positron Emission Tomography and Computed Tomography With Magnetic Resonance for Diagnosing Adult-Onset Still's Disease
Source: Front Med (Lausanne). 2020 Oct 22;7:544412. doi: 10.3389/fmed.2020.544412 (PMC7649810; doi:10.3389/fmed.2020.544412)
Supplement: Supplementary file 1 [file Table_1.docx]

**Supplementary material**

**Table 1 Suppl mat.** Correlation between Pouchot’s score and SUV ratios (spleen- and bone marrow- to liver).

| **Correlation** | **Pouchot**  **And**  **SUVmin spleen/liver** | **Pouchot**  **And**  **SUVmax spleen/liver** | **Pouchot**  **And**  **SUVmean spleen/liver** | **Pouchot**  **And**  **SUVmin BM/liver** | **Pouchot**  **And**  **SUVmax BM/liver** | **Pouchot**  **And**  **SUVmean BM/liver** |
| --- | --- | --- | --- | --- | --- | --- |
| **Spearman r** | 0.2235 | 0.3748 | 0.2626 | 0.0942 | 0.05992 | 0.05374 |
| **p value** | 0.4054 | 0.1526 | 0.3258 | 0.7284 | 0.8255 | 0.8433 |

Legend: BM=bone marrow; SUV=Standardized uptake value
